# Gait quality is improved by locomotor training in individuals with SCI regardless of training approach

**DOI:** 10.1186/1743-0003-6-36

**Published:** 2009-10-02

**Authors:** Carla FJ Nooijen, Nienke ter Hoeve, Edelle C Field-Fote

**Affiliations:** 1 The Miami Project to Cure Paralysis, University of Miami Miller School of Medicine, Miami, FL, USA; 2 Faculty of Human Movement Sciences, Research Institute MOVE, VU University, Amsterdam, The Netherlands; 3 Department of Physical Therapy, University of Miami Miller School of Medicine, Miami, FL, USA

## Abstract

**Background:**

While various body weight supported locomotor training (BWSLT) approaches are reported in the literature for individuals with spinal cord injury (SCI), none have evaluated outcomes in terms of gait quality. The purpose of this study was to compare changes in measures of gait quality associated with four different BWSLT approaches in individuals with chronic motor-incomplete SCI, and to identify how gait parameters differed from those of non-disabled (ND) individuals.

**Methods:**

Data were analyzed from 51 subjects with SCI who had been randomized into one of four BWSLT groups: treadmill with manual assistance (TM), treadmill with electrical stimulation (TS), overground with electrical stimulation (OG), treadmill with locomotor robot (LR). Subjects with SCI performed a 10-meter kinematic walk test before and after 12 weeks of training. Ten ND subjects performed the test under three conditions: walking at preferred speed, at speed comparable to subjects with SCI, and with a walker at comparable speed. Six kinematic gait quality parameters were calculated including: cadence, step length, stride length, symmetry index, intralimb coordination, and timing of knee extension.

**Results:**

In subjects with SCI, all training approaches were associated with improvements in gait quality. After training, subjects with SCI walked at higher cadence and had longer step and stride lengths. No significant differences were found among training groups, however there was an interaction effect indicating that step and stride length improved least in the LR group. Compared to when walking at preferred speed, gait quality of ND subjects was significantly different when walking at speeds comparable to those of the subjects with SCI (both with and without a walker). Post training, gait quality measures of subjects with SCI were more similar to those of ND subjects.

**Conclusion:**

BWSLT leads to improvements in gait quality (values closer to ND subjects) regardless of training approach. We hypothesize that the smaller changes in the LR group were due to the passive settings used for the robotic device. Compared to walking at preferred speed, gait quality values of ND individuals walking at a slower speed and while using a walker were more similar to those of individuals with SCI.

## Background

Spinal cord injury (SCI) frequently results in paralysis with subsequent dependence on a wheelchair for mobility. Recovery of walking function is one of the main aspirations of these individuals [1]. Different forms of locomotor training are currently available for individuals with SCI. One of the most widely used techniques is body weight supported locomotor training (BWSLT), wherein a harness/overhead lift system provides partial support to decrease loading on the lower extremities. During BWSLT on the treadmill, leg movements are often manually assisted to aid stepping. According to previous studies, BWSLT can improve the walking ability of individuals with chronic motor incomplete paraplegia or tetraplegia [2-5].

In addition to manual assistance, there are other approaches available to assist stepping. BWSLT can be aided by the use of functional electrical stimulation (FES) while walking on the treadmill or during overground walking. Despite the emphasis in the literature on the use of treadmill-based BWSLT, there is evidence from individuals with both acute [6] and chronic SCI [7] that training overground may be just as effective as training on the treadmill, while requiring less equipment. FES is used to activate the muscles or to elicit a spinal reflex response to promote movements. Previous studies have shown that the use of FES alone can improve gait in individuals with SCI [8-11]. The few studies which combined the two techniques (BWSLT and FES) in individuals with incomplete SCI were successful in improving walking speed [12,13]. Postans et al. [13] also found improvements in stride length, cadence, and gait quality (the latter based on observational methods). Field-Fote and Tepavac [14] identified improvements in intralimb hip-knee coordination associated with this training approach.

Another approach to assist stepping is combining BWSLT on the treadmill with robotic assistance. In subjects with incomplete SCI this form of locomotor training has been shown to increase walking speed [15,16]. Despite the many studies that have explored the benefits of different forms of locomotor training in individuals with SCI, it remains unclear whether one training approach is superior for improving walking function in individuals with SCI [17].

The primary purpose of this study is to quantify and compare changes in gait quality associated with four different forms of BWSLT in individuals with chronic motor-incomplete SCI. This study is part of a larger project in which a variety of outcomes associated with the locomotor training approaches are assessed. In 2005, a preliminary report of the walking-related outcomes of this study was published [7] based on an interim data analysis. While the sample size at that time was too small to have sufficient power to detect between-group differences, the data indicated that all forms of BWSLT studied were associated with improvements in walking speed. Increases in step length were found in the groups that trained on the treadmill in combination with either manual assistance or FES, and improvements in step symmetry were found in the groups that trained on the treadmill with either manual or robotic assistance. In the current account the final results and between-groups comparisons concerning the quality of gait are reported. We hypothesized that all training approaches would be associated with improvements in gait quality.

A secondary purpose of this article was to compare the gait quality of individuals with SCI to that of non-disabled (ND) individuals. Most individuals with chronic motor-incomplete SCI need a walker to be able to walk overground. Walking with an assistive device can reduce walking speed, cadence, step length, and stride length [18-23]. Furthermore, in a biomechanical analysis Alkjaer et al. [24] showed that walking with a walker can change the coordination of ND individuals. In ND individuals, we investigated the effects of walking at a speed comparable to individuals with SCI, and of walking at this comparable speed while using a walker, to enable a more accurate comparison between ND subjects and subjects with SCI.

## Methods

### Subjects

Subjects with chronic, motor-incomplete SCI were recruited from the research subject volunteer database at The Miami Project to Cure Paralysis for participation in a locomotor training study. A total of 75 subjects participated over a 5-year period. Inclusion criteria were chronic SCI defined as injury sustained at least one year prior to enrollment in the study, the ability to rise from sitting to standing with at most moderate assistance (50% effort) of one other person, the ability to advance overground using an assistive device, and damage to the spinal cord at or above the level of T12. Individuals with injury below T12 were included only if intact lower motor neuron function could be confirmed by brisk reflex responses to quadriceps, tibialis anterior, and soleus reflex testing. Exclusion criteria were current orthopedic problems, history of cardiac conditions, and presence of active hip pathology that could be aggravated by the training (e.g. severe osteoarthritis, heterotopic ossification). All subjects were medically cleared by the study physiatrist prior to participation. In addition, 10 ND subjects with no known orthopedic or neurological deficits were included for comparison of gait parameters. All subjects gave written informed consent according to the guidelines established by the Office of Human Subjects Research at the University of Miami, Miller School of Medicine.

### Training

Subjects with SCI were stratified into one of four levels based on their pre-training lower extremity strength as measured by lower extremity motor score (LEMS). The LEMS represents the sum of the scores on the manual muscle strength test for five lower extremity key muscles as defined in the international standards for neurological classification of spinal cord injury [25]. The stratification levels were Stratum 1 = LEMS of 1 - 10; Stratum 2 = LEMS of 11- 21; Stratum 3 = LEMS of 22 - 32; Stratum 4 = LEMS of >32. Subjects within each stratum were then randomly assigned to one of four training groups. The training groups were: (1) BWSLT on the treadmill with manual assistance for stepping (TM), (2) BWSLT on the treadmill with peroneal nerve stimulation to assist stepping (TS), (3) BWSLT overground with peroneal nerve stimulation (WalkAide2™, Hanger Orthopedic Group, Inc., Bethesda, MD; OG), and (4) BWSLT on the treadmill with assistance of a locomotor robot (Lokomat, Hocoma AG, Zurich, Switzerland; LR). The amount of weight support was adjusted within and between sessions as needed to prevent excessive knee flexion during stance phase or toe dragging during swing phase. Support was maintained at or below 30% (with the exception of a few subjects who needed more support during the first week of training), as this level of support is associated with gait kinematics which resemble unsupported walking [26]. Subjects in the first three groups (TM, TS, and OG) were encouraged to walk at their maximum possible speed at which step kinematics were acceptable (no toe dragging, an adequate knee flexion during swing phase, adequate knee extension at initial stance phase, etc). Subjects in the TM group received assistance for stepping based on guidelines recommended by Behrman and Harkema [27]. Subjects in the TS group received bilateral stimulation (Digitimer Ltd, Hertfordshire, UK) to the common peroneal nerve at a stimulus intensity intended to elicit a flexion withdrawal response according to procedures we have previously reported [12,14]. Subjects in the LR group walked at a speed of 2.6 km/h and speed was increased by 0.16 km/h each week with a goal of reaching the maximum device speed of 3.2 km/h. The robotic training protocol was one of passive mechanical guidance as the option to decrease the guidance forces (allowing the subject to move against the machine) was not available at the time the study was initiated. All subjects were allowed to rest as needed during the training sessions. Subjects trained 5 days per week for 12 weeks in daily sessions of 60 minutes (15 minutes of subject preparation, 45 minutes of training). None of the subjects was involved in other training activities. For more details about the training procedures see published preliminary report [7].

### Testing

Subjects with SCI performed a 10-meter walk test. During this test, kinematic data were captured within the central 6 meters of the walkway using an 8-camera infrared system (Vicon Peak™, Englewood, CO), with a 60 Hz capture rate. A total of 21 reflective markers were placed bilaterally at the lateral malleoli, 5^th ^ray metatarsal-phalangeal joints, heels, lateral knee joints, greater trochanters, anterior superior iliac spines, shoulders, elbows, and wrists as well as at C7, T10, and the sacrum. Subjects with SCI walked across the walkway five times with the instruction to walk at their fastest comfortable walking speed, and were allowed to rest between bouts. Comfortable walking speed was chosen since we believe this is most representative of everyday performance, and therefore reflects the real-world relevance of training-related changes in function. Subjects used the upper extremity assistive devices, and if necessary lower extremity orthotic devices, with which they were most familiar. Subjects used the same assistive device during the initial and final test sessions. Walk tests were performed without support for body weight or assistance for stepping. Analysis of the kinematic data was performed by two of the authors (CN and NH) who were not otherwise involved in the study.

ND subjects performed the 10-meter walk test in three different conditions on a single occasion: (1) at their preferred walking speed (PS), (2) at a walking speed comparable to subjects with SCI (0.3 m/s; CS), and (3) at a walking speed comparable to subjects with SCI (0.3 m/s) while using a walker (WCS). Each subject performed three trials of each condition, which is thought to be sufficient as little variation is expected in the walking performance of ND subjects.

### Data analysis

Kinematic data were filtered using a low-pass Butterworth filter (cutoff frequency= 6 Hz). A total of six parameters related to gait quality were calculated from the kinematic data: cadence (CAD), step length (STEP), stride length (STRIDE), symmetry index (SI), intralimb coordination (ACC), and timing of knee extension onset within the hip cycle (TOK).

CAD (steps/minute) was determined by the total number of steps divided by the time needed to complete these steps. STEP (m) of the leading leg was defined as the distance between two consecutive contralateral heel strikes. The five longest steps of each leg were selected out of all trials and these five steps were averaged for statistical analysis. The stronger leg was operationally defined as the leg that, on average, made the longest steps during the initial test for subjects with SCI, and during walking at preferred walking speed (PS) for ND subjects. The opposite leg was defined as the weaker leg. Step lengths were normalized to leg length. Leg length was calculated by adding the segmental lengths of thigh, shank, and the distance of the malleoli to the floor during stance phase of gait. STRIDE (m) was based on the five longest steps of each leg. The normalized lengths of the steps that preceded the five longest steps were added to the normalized longest step lengths for both the stronger and weaker leg.

Bilateral step symmetry was calculated using the symmetry index [28] according to equation 1:

(1)

where *SLs *is step length of the stronger leg and *SLw *is step length of the weaker leg.

A SI-value of zero represents perfect symmetry between legs. Absolute SI values were used for statistical analysis.

Intralimb coordination was defined as the ability to produce a consistent intralimb coupling relationship between knee angle and hip angle over multiple step cycles. This measure offers insights into the organization of control mechanisms underlying the coordination of walking [14,29]. The knee angle was defined as the angle formed by thigh and shank segments, and the hip angle was defined as the angle formed by trunk and thigh segments. Overall variability in the relationship between knee angle and hip angle was represented by the angular component of the coefficient of correspondence (ACC) which was calculated by using the vector coding technique [29]. An ACC value closer to 1 represents a greater consistency between knee-hip cycles. ACC values were calculated for both the stronger and weaker leg.

Timing of knee extension onset within the hip cycle was calculated to determine if the knee-hip coordination pattern in subjects with SCI is comparable to that of ND subjects. This is necessary since consistent intralimb coupling (i.e. high ACC values) of subjects with SCI do not necessarily represent a movement pattern that resembles that of ND subjects. The hip cycle was defined as the time from onset of hip flexion to the onset of the subsequent hip flexion. Within this normalized cycle, the phase value of the first knee extension onset (i.e. that occurring at approximately mid-swing) was calculated [14]. For the subjects with SCI, values closer to those obtained for ND subjects were considered representative of better gait quality.

### Statistical analysis

The statistical analysis of STEP and STRIDE data was based on the average of the five longest steps of each leg. For all other variables, the average of all trials was used for analysis. Statistical analysis was performed using SPSS version 17.0. The level of significance was set at *p *< 0.05. A two-way repeated measures analysis of variance (ANOVA) was performed to compare gait parameters. The between-subject factor was group (TM, TS, OG, and LR) and the within-subject factor was testing session (initial and final). Training, group, and interaction effects were further analyzed using post hoc tests with Bonferroni correction.

A one-way repeated measures ANOVA was used for analyzing the data of the ND subjects with condition (PS, CS, and WCS) as a within-subject factor. Condition main effects were further analyzed using post hoc tests with Bonferroni correction.

Finally, a one-way ANOVA was used to compare both the initial and final testing sessions of subjects with SCI with the WCS condition of ND subjects.

For the consideration of sphericity in all analyses, the Huyn-Feldt corrected value was used if Greenhouse-Geisser epsilon was larger than 0.75. Otherwise, the Greenhouse-Geisser corrected value was used to correct degrees of freedom in the analysis [30]. For the repeated measures ANOVA, estimates of the effect sizes for the main and interaction effects were represented by partial η2 (_p_η2). The exact effect size (η2) was calculated for the one-way ANOVA's. The effect size describes the percentage of variance which is explained in the dependent variable by a predictor while controlling for other predictors [31].

## Results

### Subjects

A total of 51 subjects with SCI were included for analysis. Data of 24 subjects (assigned to the following groups: TM = 6, TS = 7, OG = 7, LR = 4) were excluded for analysis for the following reasons: 10 subjects were unable to achieve a kinematically identifiable step during initial testing, 10 subjects withdrew from the study for personal reasons or medical reasons not related to the study, and data collection failed due to technical difficulties for 4 subjects. Although not included in the analysis, it is noteworthy that of the 10 subjects who were unable to achieve a step during initial testing, 4 were able to take steps during the final test session.

The mean number of training sessions completed by the subjects with SCI over the 12-week training period was 50 (SD = 6.57, range = 30-58). A total of 42 subjects with SCI walked with a walker, 3 with a cane, and 6 with forearm crutches. The stratified randomization into the four different training groups resulted in the following subject distribution: TM = 13, TS = 15, OG = 11, LR = 12. Additional descriptive information can be found in Table 1. The ND subject group consisted of 5 men and 5 women with a mean age of 26.5 years (SD = 9.87, range = 22-54).

**Table 1:** Descriptive information of subjects with SCI

**Group**	**Age**	**Gender**	**Chronicity (months)**	**Level of injury**	**Group**	**Age**	**Gender**	**Chronicity (months)**	**Level of injury**
TM	23	M	13	C3	TS	21	M	36	T4
	40	M	33	C5		49	M	123	C8
	41	M	21	C4		41	F	224	C4
	45	M	61	C6		38	M	122	T4
	23	M	57	C7		63	M	12	C6
	25	M	25	C6		28	M	16	T6
	23	F	64	T5		43	M	13	C6
	61	M	27	C4		40	F	237	C7
	56	M	14	C5		50	F	33	T9
	32	M	209	T4		23	M	120	C7
	50	M	74	T1		48	M	13	C4
	56	F	58	T9		50	M	128	T4
	21	M	24	T11		20	M	21	C6
						47	M	15	C4
						31	F	109	C5
Mean	38.15		52.31		Mean	39.47		81.47	
Median				C6	Median				C7
OG	49	M	12	C4	LR	56	F	120	T3
	65	M	34	C4		34	M	137	C6
	53	F	139	T1		39	M	53	C6
	41	M	49	T1		50	M	283	C6
	59	F	24	C6		48	M	16	C5
	19	M	26	C6		48	M	18	T6
	41	M	25	T6		56	M	25	C6
	24	F	47	C6		34	M	79	T5
	56	M	49	L1		54	M	252	T8
	28	M	24	C4		42	M	35	L2
	23	M	68	C5		40	F	292	T10
						31	M	21	T9
Mean	41.64		45.18		Mean	44.33		110.92	
Median				C6	Median				T4

### Missing data

Complete data sets were available to calculate STEP, CAD and SI for all included subjects. STRIDE data were (partly) missing for six subjects with SCI as an insufficient number of strides were recorded. Complete data sets for ACC were available for 38 subjects and TOK data for 27 subjects. For ten subjects with SCI included in analysis we were not able to calculate ACC and TOK as not all angle data were available. Furthermore, for three other subjects ACC and TOK could not be determined, since an insufficient number of steps were recorded during the initial measurement. For an additional 11 subjects TOK data were (partly) missing, because the knee extension or hip flexion maxima could not be determined from the angle data.

### Training effects in subjects with SCI

No significant between-group differences were found for any of the parameters, indicating that the gait parameters of interest were comparable between the different training groups, both before training and after training. Therefore pooled data of the subjects with SCI were used to assess effects of training on the selected measures of gait quality.

**Figure 1:**
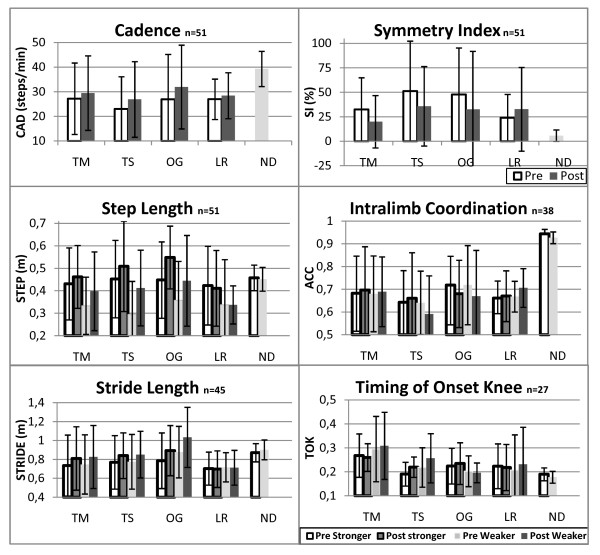
**Gait parameters of subjects with SCI before and after the four different forms of body weight supported locomotor training (TM = manual assistance, TS = peroneal nerve stimulation, OG = overground with peroneal nerve stimulation, LR = robotic assistance), and gait parameters of non disabled subjects during the condition in which they walked at a slow speed while using a walker**. Error bars represent standard deviation and n represents the number of individuals with SCI included in analysis of each parameter. CAD = cadence, SI = symmetry index, ACC = angular component of coefficient of correspondence, TOK = timing of knee extension onset.

Main effects of training were identified for cadence (F (1,47) = 14.51, *p *< 0.01, _p_η^2 ^= 0.24), step length of the stronger (F(1,47) = 14.00, *p *< 0.01, _p_η^2 ^= 0.23) and weaker leg (F(1,47) = 25.05, *p *< 0.01, _p_η^2 ^= 0.35), and stride length of the stronger (F(1,47) = 11.09, *p *< 0.01, _p_η^2 ^= 0.20) and weaker leg (F(1,47) = 20.21, *p *< 0.01, _p_η^2 ^= 0.32) (see Figure 1A, B, C). Following training, subjects in all groups were, on average, able to take more steps per minute (pre-post difference: TM = 2.3 steps/min, TS = 3.9 steps/min, OG = 5.0 steps/min, LR = 1.5 steps/min). The step and stride lengths of the subjects in the LR group did not differ more than 0.01 m between pre- and post-training, while subjects in the other groups were able to take longer steps with the stronger leg (pre-post difference: TM = 0.03 m, TS = 0.06 m, OG = 0.10 m) and weaker leg (pre-post difference: TM = 0.07 m, TS = 0.12 m, OG = 0.09 m), as well as take a longer stride with the stronger leg (pre-post difference: TM = 0.07 m, TS = 0.07 m, OG = 0.10 m) and weaker leg (pre-post difference: TM = 0.08 m, TS = 0.08 m, OG = 0.16 m).

Interaction effects were identified for step length (both strong and weak) and stride length of the weaker leg, therefore, post-hoc analyses were performed. These analyses revealed that subjects in the OG group had a significantly larger gain compared to subjects in the LR group in step length of the stronger leg (mean difference = 0.11 m, *p *= 0.01) and in stride length of the weaker leg (mean difference = 0.16 m, *p *= 0.04). Subjects in the TS group showed a significantly larger gain compared to subjects in the LR group in step length of the weaker leg (mean difference = 0.12 m, *p *= 0.02).

No main training effects were found for symmetry index, intralimb coordination (of the stronger and weaker leg), and timing of knee extension onset (of the stronger and weaker leg) (see Figure 1D, E, F). The lack of statistical improvement in intralimb coordination and timing of knee extension onset could be related to the smaller sample sizes for these parameters due to missing data (see Figure 1). However, as will be discussed in a subsequent section, timing of knee extension onset prior to training was not different from that of ND subjects; therefore changes in this measure would not be expected.

### Effects of different walking conditions in ND subjects

Main effects of walking condition were found for cadence, step length (strong and weak), and stride length (strong and weak). Compared to walking at preferred speed (PS), ND subjects took, on average, fewer steps per minute in the CS condition wherein the mean difference was 37.89 steps/minute, and fewer steps per minute in the WCS condition wherein the mean difference was 38.61 steps/minute (CAD; F(2,18) = 5.76, *p *< 0.01, _p_η^2 ^= 0.91). On average ND subjects took shorter steps with the stronger leg in the CS and WCS condition; the mean difference with the PS condition was 0.26 m for each (STEP_strong; F(2,18) = 115.72, *p *< 0.01, _p_η^2 ^= 0.93). ND subjects also took shorter steps with the weaker leg wherein mean difference with the PS condition was 0.25 m for the CS condition and 0.24 m for the WCS condition (STEP_weak; F(2,18) = 87.25, *p *< 0.01, _p_η^2 ^= 0.91). Compared to the PS condition, on average ND subjects took shorter strides with the stronger leg in both the CS and WCS condition wherein mean difference was 0.51 m for each (STRIDE_strong; F(2,18) = 87.43 *p *< 0.01, _p_η^2 ^= 0.91). ND subjects also took shorter strides with the weaker leg in the CS condition wherein mean difference was 0.54 m, and in the WCS condition wherein mean difference was 0.50 m (STRIDE_weak; F(2,18) = 104.48, *p *< 0.01, _p_η^2 ^= 0.92).

Main effects of condition were also identified for step symmetry, intralimb coordination (strong and weak), and timing of knee extension onset (strong and weak). Bilateral step symmetry was significantly smaller in the WCS condition (mean difference = 2.84) and CS condition (mean difference = 1.96) compared to the PS condition (SI; F(2,18) = 5.76, *p *= 0.01, _p_η^2 ^= 0.39). Intralimb coupling of the stronger and weaker leg was less consistent in the CS (mean difference for each leg = 0.04) and WCS condition (mean difference for each leg = 0.03) compared to the PS condition (ACC_strong; F(2,18) = 13.14, *p *< 0.01, _p_η^2 ^= 0.59 and ACC_weak; F(2,18) = 13.56 *p *< 0.01, _p_η^2 ^= 0.60). Timing of knee extension onset of the stronger leg occurred earlier in the CS condition than in the PS condition with a mean difference of 0.04% of cycle (TOK_strong; F(2,18) = 9.73, *p *< 0.01, _p_η^2 ^= 0.52). Timing of knee extension onset of the weaker leg occurred earlier in both the CS (mean difference = 0.03% of cycle) and WCS condition (mean difference = 0.04% of cycle) compared to the PS condition (TOK_weak; F(2,18) = 11.60, *p *< 0.01, _p_η^2 ^= 0.56).

### Comparison between subjects with SCI and ND subjects

Gait quality values of subjects with SCI were compared to values of ND subjects walking in the WCS condition. The WCS condition was chosen as it is most similar to the walking condition of individuals with SCI. This enables a better comparison between ND subjects and subjects with SCI, since results indicated that a reduced speed and the use of a walker can influence gait quality.

Subjects with SCI had a cadence that was significantly lower than that of ND subjects walking in the WCS condition (see Figure 1A). Subjects with SCI took on average 13.44 fewer steps/minute in the initial test, and 10.28 fewer steps/minute in the final test compared to the ND subjects (initial test; F(1,59) = 8.99, *p *< 0.01, η^2 ^= 1.88 and final test; F(1,59) = 4.96, *p *= 0.03, η^2 ^= 1.44). In the initial test subjects with SCI took steps with the weaker leg that were on average 0.12 m shorter than the steps of the ND subjects (F(1,59) = 5.9, *p *= 0.02, η^2 ^= 2.34). During the final test this difference was no longer significant (F(1,59 = 0.92, *p *= 0.34, η^2 ^= 1.02). No significant differences were found between subjects with SCI and ND subjects for step length of the stronger leg, and for stride length of both the stronger and weaker leg (see Figure 1B, C).

Bilateral step symmetry was significantly lower for subjects with SCI, compared to ND subjects during the initial test as indicated by larger SI values for the subjects with SCI (mean difference = 33.45, F(1,59) = 4.88, *p *= 0.03, η^2 ^= 11.33). After training, there was no longer a significant difference in SI between subjects with SCI and ND subjects (F(1,59) = 3.42, *p *= 0.07, η^2 ^= 8.30) (see Figure 1D).

The initial intralimb coordination values differed significantly between subjects with SCI and ND subjects for both the stronger leg (F(1,59) = 43.58 *p *< 0.01, η^2 ^= 13.45) and the weaker leg (F(1,59) = 32.09 *p *< 0.01, η^2 ^= 9.73). There remained a difference between ND subjects and subjects with SCI during the final test for both the stronger leg (F(1,59) = 26.24, *p *< 0.01, η^2 ^= 13.20) and the weaker leg (F(1,59) = 25.01, *p *< 0.01, η^2 ^= 10.08). ND subjects had a more consistent intralimb coupling during the pre (mean ACC difference = 0.27) and post test (mean ACC difference = 0.26) for the stronger limb and during the pre (mean ACC difference = 0.25) and post test (mean ACC difference = 0.26) for the weaker limb (see Figure 1E).

No significant differences between subjects with SCI and ND subjects were found for the timing of knee extension onset at the time of the initial or final test (see Figure 1F).

## Discussion

### Training effects in subjects with SCI

When selecting a locomotor training approach for individuals with chronic SCI, the therapist may decide to give primary attention to an approach that focuses on improving gait quality. In such cases the results of this study indicate that there are several options, as all four BWSLT approaches were associated with improvements in variables associated with gait quality and no significant differences among groups were found.

This is the first article with a main focus on improvements in gait quality after locomotor training in individuals with chronic SCI. Across training groups subjects with SCI showed significant improvements in cadence, step length and stride length. The data indicated that, on average, increases in step length were larger for the weaker leg compared to the stronger leg, which could be related to the (non-significant) increase in the bilateral step symmetry. The large variation observed in the step symmetry of subjects with SCI could be the reason that this increase was not statistically significant (see Figure 1D). In individuals with acute SCI Postans et al. [13] also found increases in cadence and stride length after BWSLT combined with electrical stimulation. Since individuals in that study were trained during the acute phase of SCI, it is likely that part of the observed improvements may have been due to the spontaneous recovery that occurs in the first post-injury year. Improvements in step and stride length were also found after locomotor training with electrical stimulation without body weight support [9,11]. Furthermore, in individuals with stroke, BWSLT has shown to be effective in improving gait quality [3,32-38]. Since there is a considerable variability in training protocol, intensity, and subjects among the studies, it is complicated to make a good comparison between the amounts of improvement. Therefore, more research is necessary about the specific influences of training parameters [39].

Regardless of the approach, BWSLT leads to improvements in gait quality. This conclusion is in accordance with a recent review of Merholz et al. [17] in which it was concluded that the different forms of locomotor training used in the present study are all effective in improving walking speed and capacity. However, group effects could have been masked in the current study, since there was a large variation among subjects within the different training groups for all parameters, in part because the study design was intended to include both higher and lower functioning subjects in each group. This large variation could have accounted for an overlap in outcomes between groups (see Figure 1).

Subjects who received electrical stimulation (TS and OG group) improved step and stride length to a greater extent than subjects who were trained with robotic assistance (LR group). The LR group showed no or only slight increases in step and stride length, while the other training groups improved substantially on these parameters. Also, mean changes in bilateral step symmetry tended to favor the other three training approaches above LR. It is essential to note however, that in the present study the robotic training protocol did not include the option to decrease the guidance forces and require the subject to exert effort, as this option was not available on the device at the time this study was initiated. Although subjects in the LR group were encouraged to "walk with the machine," it is likely that these subjects did not exert the level of voluntary effort that was exerted by subjects in the other groups. These results may indicate that voluntary effort is important for developing the motor skills required for improvements in gait quality. The results also suggest that BWSLT combined with passive mechanical guidance is not the preferable training approach for improving gait quality in individuals with chronic SCI. However, this training approach could be more effective for subjects with more severely impaired locomotor function. Furthermore, the use of the more active Lokomat training protocol, using software that was not available at the time this study was initiated, might lead to other results, and further research is necessary to find the optimal set of training parameters for walking with robotic assistance [40].

For all training approaches, locomotor training did not lead to a more consistent coordination between limbs and the timing of knee extension onset was not altered by training. These results are in contrast to the outcomes of a study of Field-Fote et al. [14] in which the consistency of the intralimb coordination in subjects with SCI increased and the timing of knee extension onset was earlier in the hip cycle following locomotor training. Difference in training and testing procedures between the two studies may explain differences in findings. In the study by Field-Fote et al. [14], subjects were trained and tested on a treadmill. In the present study three of the four groups were trained on the treadmill, while the gait analyses were all performed overground. According to Alton et al [41], comparison of overground and treadmill gait analyses should be avoided in patients. Significant differences in hip and knee motion variables are found between overground and treadmill walking in several studies [41-44]. The finding that timing of knee extension onset of subjects with SCI was not different from that of the ND subjects in the present study wherein testing was based on overground walking, but was different in a similar group of subjects wherein testing was based on treadmill walking [14], suggests that the walking environment influences the gait parameters related to coordination. The finding of no change in intralimb coordination in subjects who (for the most part) were trained on the treadmill but tested overground, may reflect incomplete transfer of motor learning underlying the control of coordination from the treadmill to the overground condition.

### Different walking conditions in ND subjects

When the ND subjects walked both with and without a walker (WCS and CS condition) at a speed that was comparable to that of individuals with SCI, they took fewer steps per minute and decreased the length of their steps and strides. This modification of cadence, and step and stride length is typical of ND individuals when they desire to adjust walking speed [45]. Furthermore, walking at this speed resulted in a less consistent intralimb coordination. This is in accordance with previous research in which correlations between speed and intralimb coordination were found [14,46,47]. Finally, during walking at reduced speed, the onset of first knee extension occurred later in the hip cycle. Our finding that gait quality changes when walking at reduced speeds regardless of whether a walker was used, suggests that prior studies wherein a reduction in gait quality has been attributed to the use of the assistive device [18-23], the reduced gait quality may not reflect a direct result of the use of an assistive device. Rather, it is likely that the assistive device caused the ND individuals to reduce their speed, which indirectly resulted in reduced gait quality.

In addition to the changes in intralimb coordination that accompanied walking at reduced speeds (with and without a walker) in ND individuals, walking with a walker resulted in less symmetry of bilateral stepping. For the condition in which subjects only walked at a reduced speed (without a walker), the step symmetry was comparable to that when walking at preferred speed. This indicates that the use of an assistive device can change the symmetry between the limbs.

These results suggest that when attempting to identify how the gait quality of individuals who walk at a reduced speed and require an assistive device (such as those with SCI) differs from "normal" gait, walking speed may have a greater influence on these parameters than the use of the assistive device. However, this should not be construed to suggest that the walking speed during locomotor training is the critical factor in improving walking function, as our prior work indicates that those who train at slower speeds while walking overground make improvements in walking function that are, in some cases, greater than those experienced by individuals who train at faster speeds on the treadmill [7].

### Comparison between subjects with SCI and ND subjects

Following three months of daily locomotor training, several parameters of the gait quality of subjects with SCI improved such that it became more similar to the gait quality of ND subjects. The mean difference in cadence between ND subjects and subjects with SCI during the final test was smaller compared to the mean difference during the initial test. The significant shorter step length of the weaker leg and the bilateral step asymmetry that subjects with SCI exhibited at the initial test were no longer present at the time of the final test, and these gait parameters were comparable to those of the ND subjects. Step length of the stronger leg and stride length were already comparable between subjects with SCI and ND subjects at the start of training. No differences were found for timing of knee extension onset in the hip cycle.

### Limitations

As stated previously, the large amount of variability in all outcome parameters of subjects with SCI may be responsible for the lack of significant findings related to some of the parameters of interest. The large variability is likely due to differences in the degree of injury among subjects. However, variability is a well-known and mostly insurmountable problem in studies of individuals with neuropathology.

Furthermore, a large number of subjects were excluded from the analyses either because the individual was unable to achieve a step that was kinematically identifiable, withdrew from the study, or the kinematic data set was incomplete. The lack of an intention-to-treat analysis is a limitation of this study. The number of subjects excluded due to lack of a kinematically identifiable step (n = 10) or because of incomplete kinematic data (n = 4) may have been lower if testing had been repeated on multiple days. Since individuals with SCI have a day-to-day variation in standing performance and ability to walk, a larger amount of within-subject baseline data would likely have reduced the variability in outcome parameters. In addition, some of the subjects who were unable to take any steps may have been able to make an identifiable step during at least one of the test days with repeated testing. However, given that at least three steps on each side are required to make meaningful conclusions about gait quality, and since only four out of ten of these subjects were able to take steps after training, repeated testing may not have made a large difference in the data for this subgroup of subjects.

This study assessed only locomotor training approaches that used partial support for body weight. It is not known how these results compare to outcomes of locomotor training wherein support for body weight is not provided. Results in individuals with acute SCI appear to indicate that support for body weight is not a critical factor [6]. It is also possible that a combination of treadmill-based BSWLT and overground training without body weight support may be the optimal approach [48]. However, direct comparisons must be made in individuals with chronic SCI before definitive conclusions can be reached.

## Conclusion

Regardless of training approach, gait quality improved in individuals with chronic motor-incomplete SCI following BWSLT, such that the values became more similar to those of ND individuals. The greatest improvement in gait quality was found for subjects who trained with electrical stimulation. Less improvement was found for individuals who trained with passive robotic assistance. Furthermore, when ND subjects were walking at a reduced speed and when they were using a walker, their gait quality values changed and became more comparable to those of individuals with SCI.

Based on the results of this study, therapists can be confident that practice in walking is the key element for the success of a BWSLT program wherein the goal is to improve gait quality in individuals with chronic motor-incomplete SCI. Furthermore, when comparing gait quality values of individuals who walk at a reduced speed and require an assistive device (such as those with SCI) and ND individuals, it is advised to use data in which ND subjects walk at a reduced speed while using a walking device.

## Competing interests

The authors declare that they have no competing interests.

## Authors' contributions

NH and CN performed the data analysis of all subjects, measurements of healthy subjects, statistical analysis, and drafted the manuscript. EF participated in the design, execution, and coordination of the study and assisted with drafting the manuscript. All authors read and approved the final manuscript.
